# Metagenomic analysis of viromes in tissues of wild Qinghai vole from the eastern Tibetan Plateau

**DOI:** 10.1038/s41598-022-22134-y

**Published:** 2022-10-14

**Authors:** Xiaozhou He, Xu Wang, Guohao Fan, Fan Li, Weiping Wu, Zhenghuan Wang, Meihua Fu, Xu Wei, Shuo Ma, Xuejun Ma

**Affiliations:** 1 NHC Key Laboratory of Medical Virology and Viral Diseases, National Institute for Viral Disease Control and Prevention, Chinese Center for Disease Control and Prevention, Beijing, People’s Republic of China; 2 Chinese Center for Disease Control and Prevention - Wuhan Institute of Virology, Chinese Academy of Sciences Joint Research Center for Emerging Infectious Diseases and Biosafety, Center for Biosafety Mega-Science, Chinese Academy of Sciences, Wuhan, People’s Republic of China; 3 National Institute of Parasitic Diseases, Chinese Center for Diseases Control and Prevention (Chinese Center for Tropical Diseases Research), NHC Key Laboratory of Parasite and Vector Biology, WHO Collaborating Centre for Tropical Diseases, National Center for International Research on Tropical Diseases, Shanghai, People’s Republic of China; 4 School of Life Sciences, East China Normal University, Shanghai, People’s Republic of China; 5 Shanghai Municipal Center for Disease Control and Prevention, Shanghai, People’s Republic of China

**Keywords:** High-throughput screening, Virology, Metagenomics

## Abstract

Rodents are natural reservoirs of diverse zoonotic viruses and widely distributed on the Tibetan Plateau. A comprehensive understanding of the virome in local rodent species could provide baseline of viral content and assist in efforts to reduce the risk for future emergence of rodent related zoonotic diseases. A total of 205 tissue and fecal samples from 41 wild Qinghai voles were collected. Metagenomic analyses were performed to outline the characteristics of the viromes, and phylogenetic analyses were used to identify the novel viral genomes. The virome distribution among five tissues (liver, lung, spleen, small intestine with content and feces) was also compared. We identified sequences related to 46 viral families. Novel viral genomes from distinct evolutionary lineages with known viruses were characterized for their genomic and evolutionary characteristics, including *Hepatovirus*, *Hepacivirus*, *Rotavirus*, and *Picobirnavirus*. Further analyses revealed that the core virome harbored by rodent internal tissues were quite different from the virome found in intestine and fecal samples. These findings provide an overview of the viromes in wild Qinghai voles, which are unique and the most common rodent species in the eastern Tibetan Plateau. A high diversity of viruses is likely present in rodent species in this area.

## Introduction

Zoonotic diseases comprise a significant and increasing proportion of all human infectious diseases, and pose a significant threat to human health and animal welfare^1^. Despite limited knowledge of the origins of most zoonotic viruses, primates, birds, bats, and rodents are considered major reservoirs due to their ability to host wide ranges of viruses^2–4^. Emerging viruses are well adapted to many of these reservoir host species, with little or no evidence of disease^5,6^. However, when these viruses spill over into humans, the effects can sometimes be devastating^7,8^. Rodents are distributed worldwide and interact with humans and other animals in many ways. The human infection risk is often positively correlated with the abundance of the rodent host population^9,10^. Continuously expanding human activities have promoted interactions between humans and infected rodents, and thus the opportunities for zoonotic transmission have been amplified^11^.

Rodents are the largest group of mammals worldwide with approximately 2277 species recognized, accounting for over 40% of all mammals, including mice, rats, voles, and many others^7,12^. Rodents vector more than 60 human infectious diseases, such as members of the *Bunyaviridae*, *Reoviridae*, *Picornaviridae*, *Coronaviridae*, and *Flaviviridae* families^13–15^. The abundance of rodent species, together with their fast-paced lives, population dynamics, variety of habitats, and close contact with domestic animals and wildlife, makes them efficient amplifiers, spreaders, and transmitters of viruses between animals and humans^12,16^. Moreover, rodents play important roles in the natural circulation of vector-borne viruses^17^. Therefore, virus discovery studies in wild rodent populations may benefit public health.

The development of high-throughput sequencing and metagenomic approaches has led to a rapid increase in knowledge on viral diversity^18,19^. A wide diversity of viral sequences in rodents have been identified, many of which were previously unknown species or variants, such as novel picornaviruses and hepaciviruses^13,20^. A range of known and novel viral pathogens, such as members of the families *Arteriviridae*, *Arenaviridae*, *Flaviviridae*, *Hantaviridae*, *Herpesviridae*, and *Phenuiviridae*, have been identified in the lungs of rodents from mainland Southeast Asia^17^. Systematic virome in rodents collected from different locations of China were surveyed, which has greatly increased our knowledge of the viral community in rodents^11,16^. Much data has been provided by rodents virome surveillance programs, which provide insight into existing viral populations and their ecology in ecosystems before they emerge into human populations causing large-scale outbreaks^7,21^.

The Tibetan Plateau, also known as the Qinghai-Tibet Plateau, located in western China, is the highest and one of the most extensive plateaus in the world, with an average elevation of more than 4000 m. This vast region consists mostly of plateau and grassland supporting well-developed animal husbandry^22^. The plateau is also an epidemic area of diverse zoonoses, including bacteria (plague), viruses (e.g., astrovirus, influenza virus, tick-borne encephalitis virus, and coronaviruses), and parasites (e.g., *Echinococcus multilocularis*) disease, particularly for agriculture and animal husbandry workers in direct or indirect contact with infected animals^23–30^. The plateau provides ideal habitats for small rodents such as house mouse, pika, vole, and marmot, because unfettered access to shelter and food sources provides ample opportunity for them to thrive^31^. The continuous maintenance and spread of their populations, aided by a rapid and prolific breeding cycle, are key factors in the high levels of interaction with wildlife and livestock^9,12^. These rodents live in close association with livestock, poultry, and humans, offering numerous opportunities for cross-species viral transmission through their urine, feces, or their arthropod ectoparasites such as ticks, mites, and fleas into the environment^5,32^. Qinghai voles (*Microtus fuscus*, family Cricetidae) are unique and the most common rodent species in high mountain grazing areas, particularly in southern Qinghai Province and northeast Sichuan Province^33^. Viruses carried by Qinghai voles in this region have been poorly investigated, although they potentially pose a major risk to public health. Therefore, it is necessary to enhance our knowledge of the virome diversity in Qinghai voles to facilitate future prevention and control of emerging zoonotic diseases.

## Results

### Overview of the viromes

**Figure 1:**
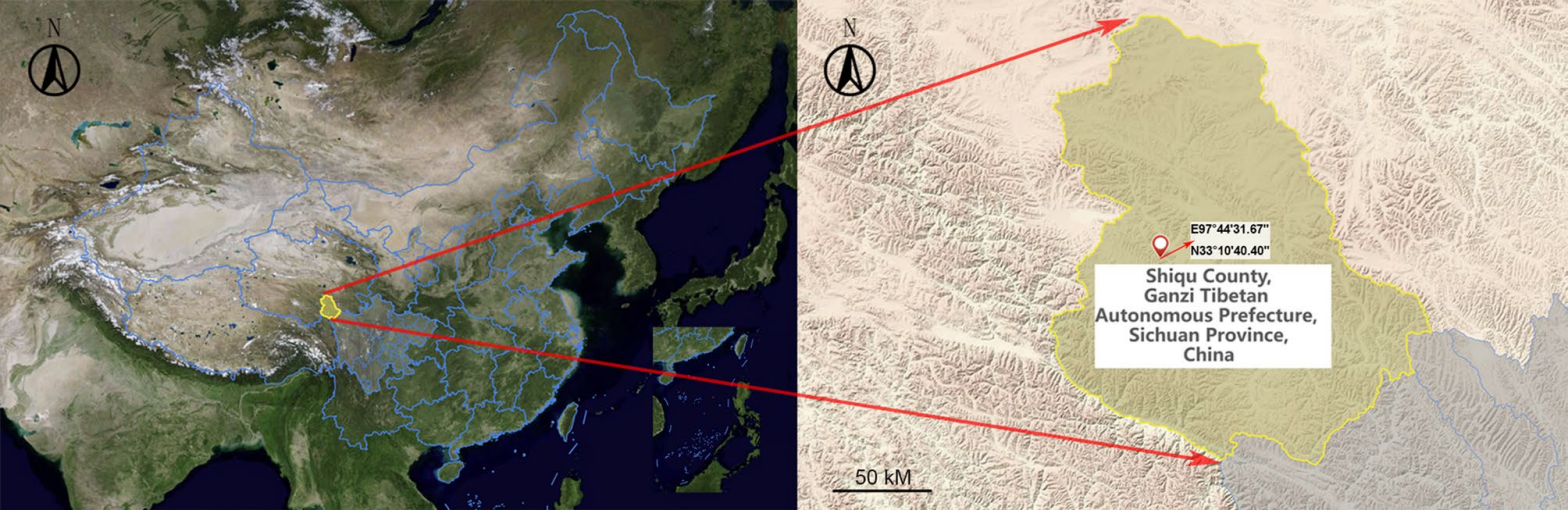
Satellite map (left) and topographic map (right) of the rodent collection area on the eastern Tibetan Plateau of China. Shiqu county is highlighted yellow, and Sichuan province is marked in light gray, the geographic coordinate of collection site (E97°44′3E.67", N33°10′40.40") is marked on the topographic map. The map was generated by SuperMap (http://www.supermapol.com/).

In all, 41 wild Qinghai voles were collected from pasture habitats located on the eastern Tibetan Plateau, China (Fig. 1). Tissue samples from liver, lung, spleen, small intestine (with content), and feces (large intestinal content) of each vole were disrupted, and viral RNA was extracted. The RNA samples were combined into 20 pools of equal quantities according to sample type (Supplementary Table S1). Overall, 729,234,124 paired-end reads with an average of 150 bp in length were obtained from 20 libraries, yielding an average of 36.5 M (95% CI: 35.2–37.8 M) reads per pool. After filtering by fastp, 98.3–99.5% of raw reads were retained, and 722,035,886 clean reads were used for further analyses, of which 67.5% mapped to the host genome. Reads classified as cellular organisms (including eukaryotes, bacteria, and archaea) and those with no significant similarity to any amino acid sequence were discarded, leading to 1,472,071 reads best matched with viral sequences, accounting for 0.31% of total clean reads. Due to the presence of numerous transcripts from the hosts and other organisms, most pools had low levels of viral RNA. The percentage of virus-associated reads in each pool was 0.05–2.47% (Supplementary Table S1).

**Figure 2:**
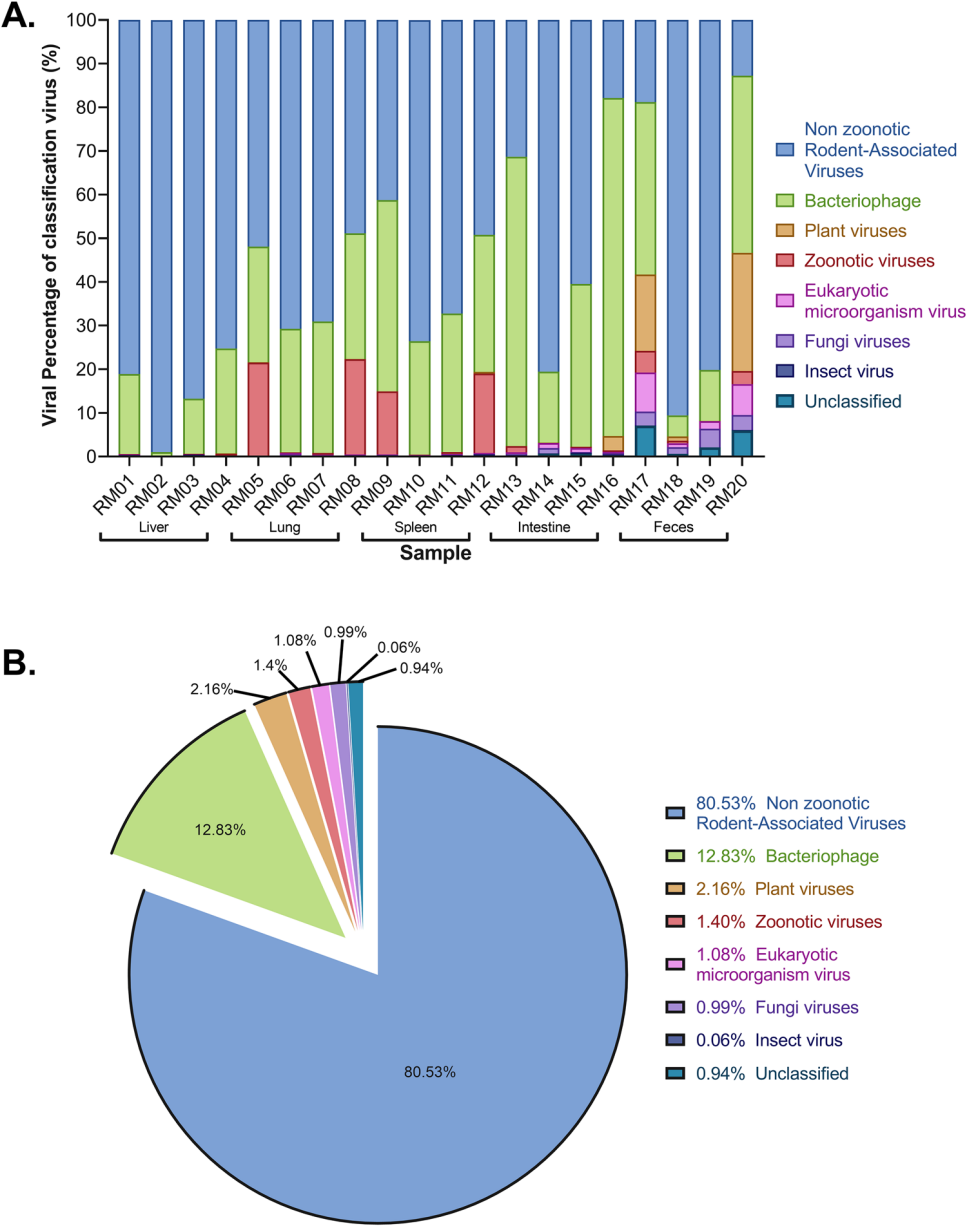
Proportion of viral sequence reads with BLASTX hits to the specified virus families. (**A**) Proportion in each library. The y-axis is the percentage of viral reads distribute to each classification, or that were unclassified viruses. The sample ID is shown on the x-axis. The percentage of reads was determined based on the raw number of viral-related reads. (**B**) Proportion in total viral reads.

A wide range of DNA and RNA virus groups were covered by the sequence reads. Virus-associated reads were assigned into 46 families of double-stranded (ds)DNA viruses, dsRNA viruses, retro-transcribing viruses, single-stranded (ss)DNA viruses, and ssRNA viruses (positive- and negative-strand viruses) in the virus root. Based upon natural host of each virus, we classified 13 families of these viruses as vertebrate-associated viruses (6 zoonotic viruses and 7 non-zoonotic rodent associated viruses), 11 as bacteriophages, 10 plant viruses, 6 as fungal viruses, 1 as an insect virus, 5 as eukaryotic microorganism (protozoa and algae)-related viruses and a group of unclassified viruses (Supplementary Fig. S2 and Table S2). An overview of the classified and unclassified viral reads is shown in Fig. 2A.

The largest proportion of the virus-classified sequences was related to vertebrate viruses, with 81.93% of the total viral reads, which included zoonotic viruses (1.4%) and non-zoonotic rodent associated viruses (80.53%). Among them, viral sequences related to ssRNA positive-strand viruses in the *Picornaviridae* were abundant, comprising 65.6% of the total virus-like sequence reads. The dsDNA viruses were predominantly bacteriophages such as *Ackermannviridae*, *Autographiviridae*, unclassified bacteriophages and nine other families, accounting for 12.8% of the total viral reads. In addition, 4.3% of the viral sequences were related to insect viruses (0.06%), plant viruses (2.16%), fungus viruses (0.99%), and eukaryotic microorganism viruses (1.08%) (Fig. 2B). Detection of these viral sequences may be due to food consumption. In addition to the family assigned reads, 0.94% of total viral reads were identified as unclassified RNA viruses, including diverse bunyavirale*s*, picornavirales, riboviria, and environment-related viruses. Except for unclassified virus and bacteriophage (11 families), the top 10 most widely distributed families of viruses were *Picornaviridae*, *Flaviviridae*, *Retroviridae*, *Picobirnaviridae*, *Solemoviridae*, *Arteriviridae*, *Mitoviridae*, *Mimiviridae*, *Phycodnaviridae*, and *Reoviridae*. Samples of wild Qinghai voles had marked virus diversity.

### Virome distribution in different tissues

**Figure 3:**
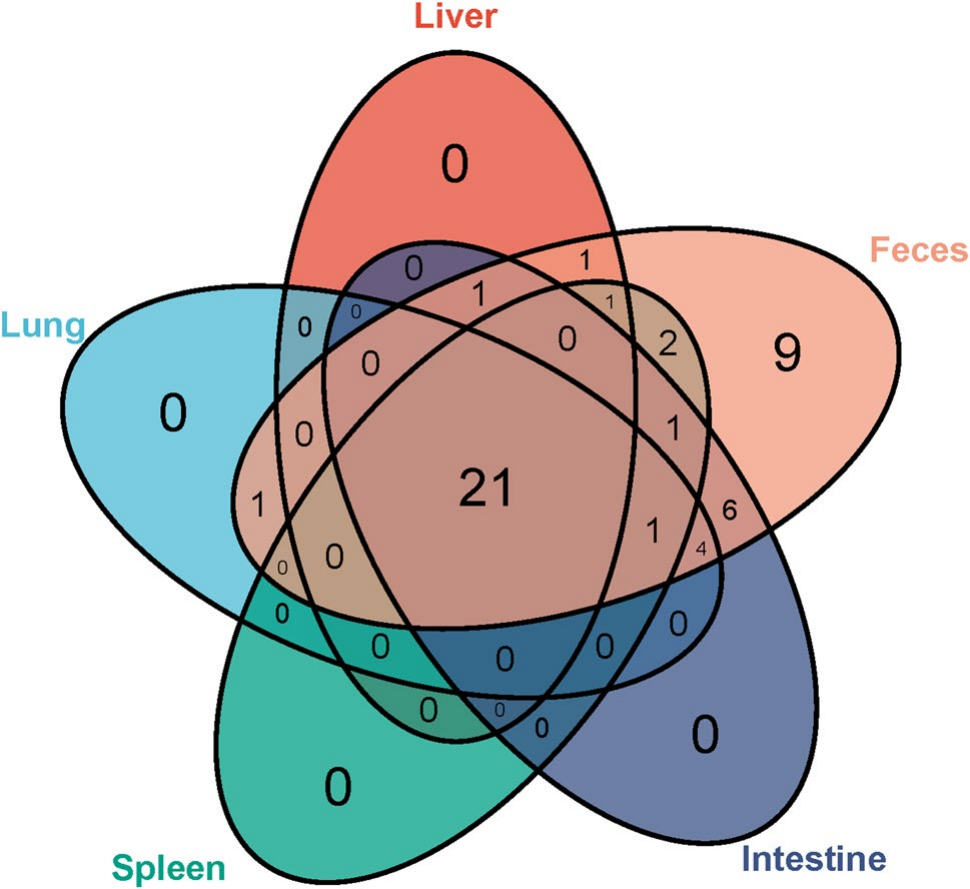
Venn diagram of viral families shared in the five tissues. The numbers represent viral families found in each tissue. A total of 48 viral taxa were analyzed and displayed, which included 46 viral families, one unclassified virus and one unclassified Bacteriophage.

Venn analyses revealed that 21 viral families, including *Picornaviridae*, *Flaviviridae*, *Retroviridae*, and *Picobirnaviridae*, were distributed in the five tissues (Fig. 3, Supplementary Table S3). However, the Venn diagram demonstrated that nine viral families—such as *Coronaviridae*, *Parvoviridae*, *Hypoviridae*, *Autographiviridae* and five plant viruses—were unique to feces, which indicated that these viruses have compartment specificity. In addition, six viral families were shared between intestine and feces. The other 12 viral families were found in at least two tissues, one of which was a fecal sample.

**Figure 4:**
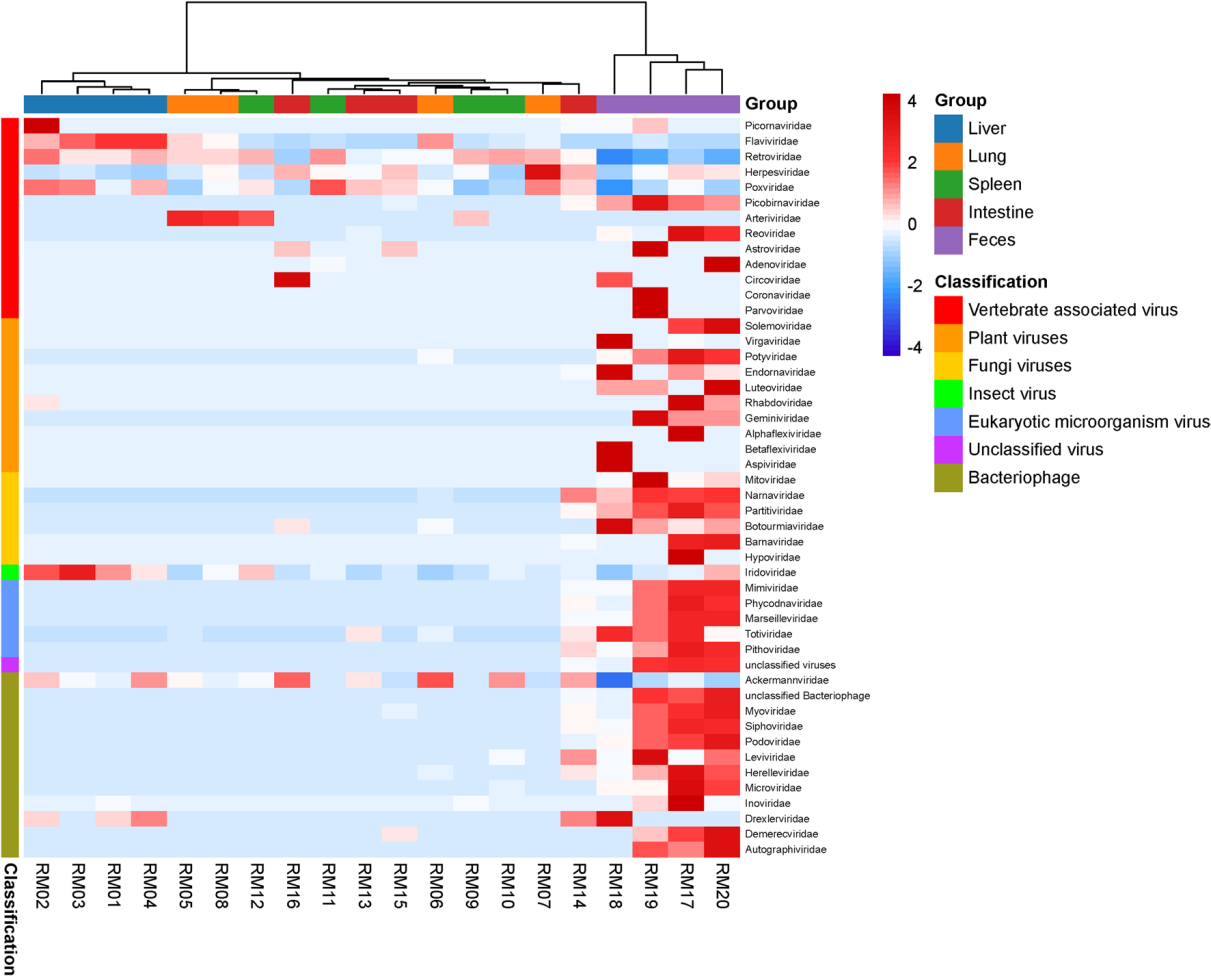
Heatmap based on the distance matrix calculated by the Euclidean distance method according to the normalized number of reads belonging to each viral family in 20 pools. X axis shows sample names, and the Y axis the names of viral families. Red to blue, highest to lowest abundance of viral reads according to viral family. The hierarchical clustering is based on the Euclidean distance matrix calculated from the normalized read count. A total of 48 viral taxa were analyzed and displayed, which included 46 viral families, one unclassified virus and one unclassified Bacteriophage. The heatmap was generated by Hiplot (v0.2.0, https://hiplot.com.cn).

The results suggest that liver and feces act as major reservoirs for diverse viruses in wild Qinghai voles, accounting for 55.3 and 26.1% of total viral reads, respectively. To detect differences in virome structures among the samples, taxonomic heatmap and hierarchical cluster analyses were conducted based on the normalized viral reads number. A heatmap of all reads to the sequences of the 46 viral families, unclassified virus and unclassified Bacteriophage identified in this study is shown in Fig. 4. For instance, in liver, *Picornaviridae*, *Flaviviridae*, *Iridoviridae*, and *Poxviridae* were abundant. In lung, *Herpesviridae* and *Arteriviridae* were the most abundant virus families. The most abundant viral family in spleen was *Retroviridae*. In intestine, *Ackermannviridae* and *Circoviridae* were abundant. However, 37 viral families and unclassified virus were abundant in feces. Compared to the other tissues, liver and feces samples clustered together separately, which indicated a closer correlation of virome structures. Overall, our results revealed significant differences in virus composition and abundance among tissues.

### Characteristics of selected zoonotic viruses

By characterizing host traits and transmission routes, non-vertebrate-associated viral reads, bacteriophages, and unclassified viruses reads described previously were removed. The remaining 1,206,124 viral reads (approximately 81.93% of the total viral reads) were assigned into 13 vertebrate-related viral families. Viral reads from the families *Picornaviridae*, *Flaviviridae*, *Retroviridae*, *Picobirnaviridae*, *Arteriviridae*, *Poxviridae*, and *Herpesviridae* were widely distributed in tissues, in different abundances. The families *Reoviridae*, *Adenoviridae*, *Astroviridae*, *Coronaviridae*, *Circoviridae*, and *Parvoviridae* were found in few tissue types. Analyses of the virus reads distribution showed that 965,703 reads (65.5% of total viral reads) exhibited sequence similarity to *Picornaviridae*, accounting for a major portion of the total virus reads (Supplementary Table S2). Other mammalian virus sequences in order of sequence read abundance were *Flaviviridae* (8.27%), *Retroviridae* (3.36%), *Picobirnaviridae* (3.27%), and other families, accounting for 1.43% of viral reads. These viruses belonged to a genus or family known to cause human or animal infection were confirmed by PCR amplification using specific primers. All these viral reads were extracted from each dataset and submitted to de novo assembly by SPAdes software, length and depth of assembly contigs were shown in Supplementary Table S4. Blast results indicated that these genomes showed low nucleotide (nt) or amino acid (aa) similarity to known genome sequences in the GenBank database. We characterized some of these full or near-full genome sequences and compared them to their closest relatives by phylogenetic analyses.

#### Picornaviridae

**Figure 5:**
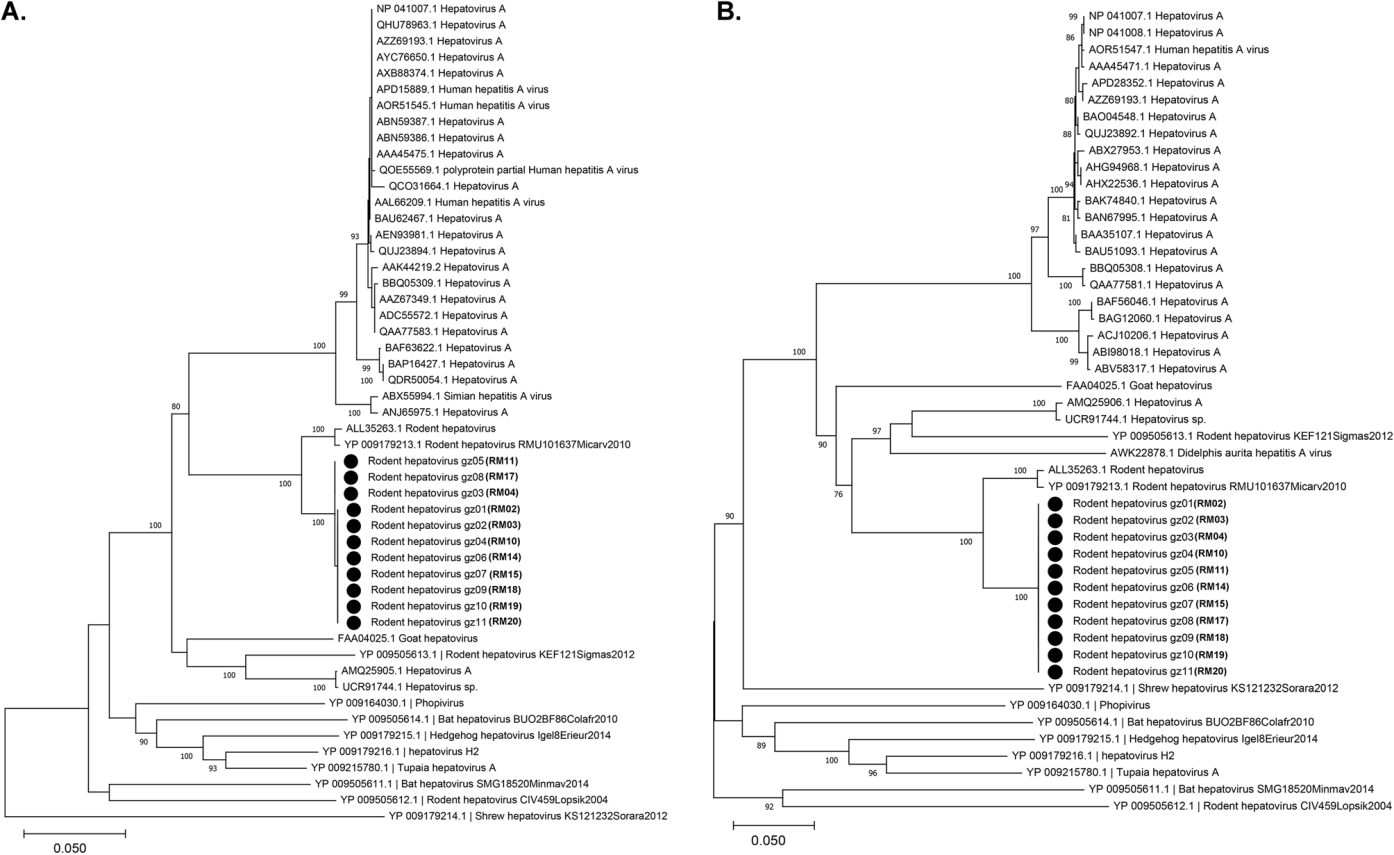
Phylogenetic relationships of hepatovirus variants based on analyses of the P1 protein (**A**) and 3CD protein (**B**). Branch lengths are drawn to a scale of aa substitutions per site. Numbers above individual branches indicate bootstrap support, only values > 80% are shown. Vole hepatovirus variants are marked by a black dot, sample ID were labeled in parentheses.

Eleven near-complete genomic sequences for Picorna-like viruses were identified in all tissues except lung. Reads related to the *Picornaviridae* family comprised the largest proportion of viruses, particularly in liver (85.9%), small intestine (51.5%), and feces (45.7%) samples. The distribution of these picorna-like viruses among tissues was similar to picornavirus, which infect the liver and are transmitted by the fecal-oral or blood route^34,35^. Overall, these 11 genome sequences of picorna-like virus were retrieved from the pools and were of 7448–7640 bp. Using NCBI’s ORF finder, it was predicted that both genomes had a single ORF encoding a polyprotein, similar to the genome structure of *Hepatovirus*^13,36^. The nt identity between contigs ranged from 99.0 to 99.9%, showing great similarity. Moreover, sequence similarity and phylogenetic analyses indicated that all contigs clustered with rodent hepatovirus. Therefore, these genomes were classified into the genus *Hepatovirus* (Fig. 5). BLASTn search revealed that these sequences were closely related to rodent hepatovirus (KT452641.1, *Myodes glareolus*, collected in Germany in 2011) with nt sequence identities between 82.74 and 82.76% (Supplementary Table S4). BlastX analyses revealed that these contigs were 91.83–91.88% similar at the aa level to their closet relative polyprotein, that of rodent hepatovirus (YP_009179213.1, *Microtus arvalis*, collected in Germany in 2010). According to the ICTV criteria, the divergence of members of hepatovirus species ranges from 0.18 to 0.40 for the P1 region and 0.19–0.49 for the 3CD region^37^. The distance was 0.03–0.04 for the P1 region and 0.07 for the 3CD region between these contigs and rodent hepatovirus. Therefore, these contigs were proposed to be novel variants of rodent hepatovirus.

#### Flaviviridae

**Figure 6:**
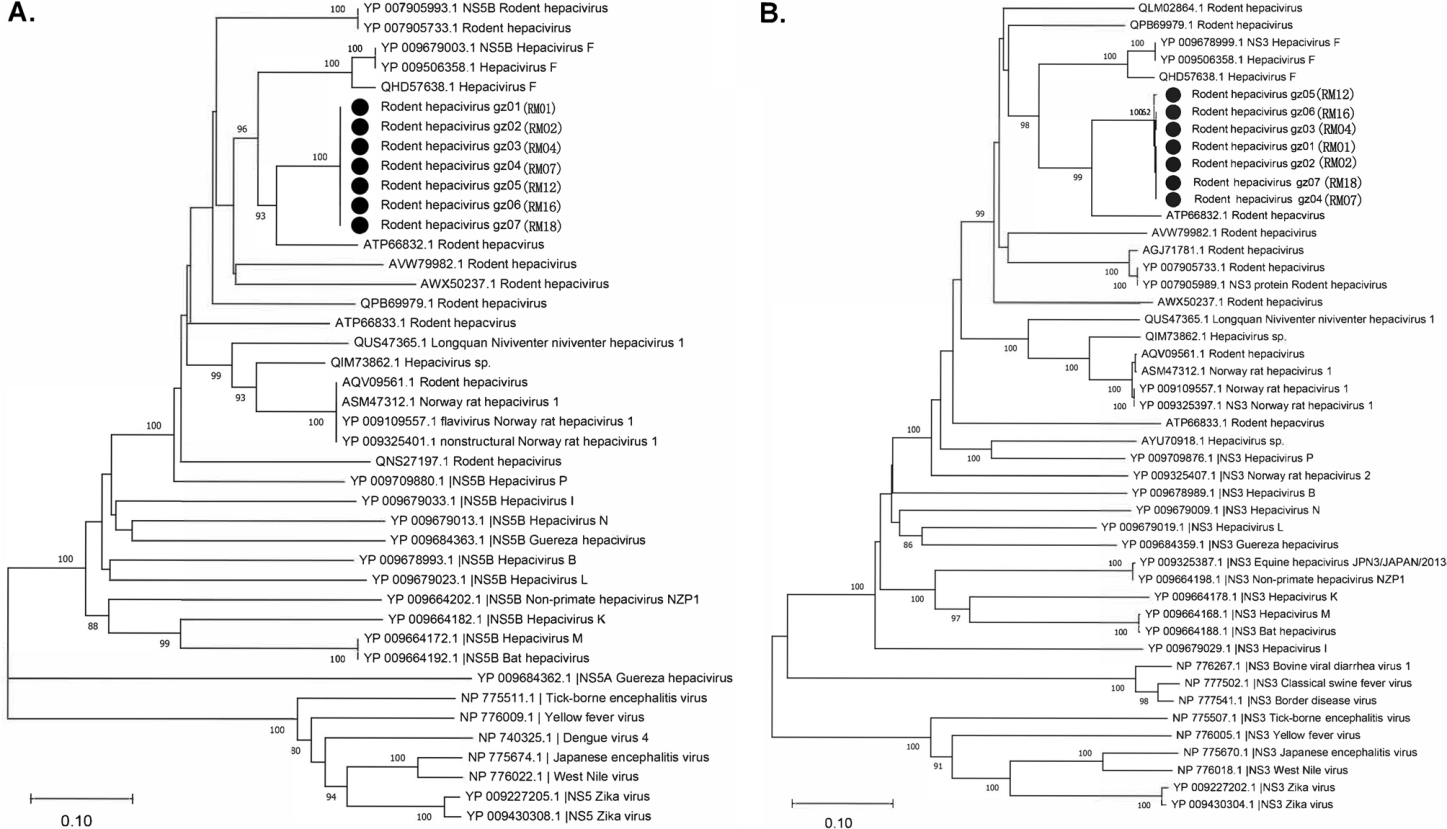
Phylogenetic analyses of hepacivirus variants based on the NS5B (**A**) and NS3 (**B**) protein. Branch lengths are drawn to a scale of aa substitutions per site. Numbers above individual branches indicate bootstrap support, only values > 80% are shown. Hepacivirus variants are marked by a black dot, sample ID were labeled in parentheses.

In all, 121,679 reads were assigned to the family *Flaviviridae* (Supplementary Table S2), being found in almost all tissues. Such a broad distribution indicates diverse modes of potential transmission, such as vertical and fecal-oral. Seven near-complete genomic sequences were identified in samples (three in liver, one in lung, one in spleen, and two in feces) by de novo assembly, with a length of 8617–8625 bp. These sequences shared 99.2–99.9% identity to each other. Sequence analyses using NCBI ORF finder revealed a single ORF translated into a polyprotein, with a genome structure similar to typical *Flaviviridae*^16,38,39^. These contigs were subjected to PCR confirmation and whole-genome phylogenetic analyses. All contigs were assigned to a clade in the genus *Hepacivirus* with various sequence similarities to rodent hepaciviruses collected from *Neodon clarkei* in Tibet, China in 2014. The contigs showed 75.63–75.73% nt identity and 82.83–88.90% aa identity with rodent hepacivirus (Fig. 6 and Supplementary Table S4). According to the ICTV guidelines, hepaciviruses with < 0.25 aa p-distances in the conserved region of NS3 and 0.3 in the NS5B region belong to the same species^40^. Because the NS5B and NS3 region p-distances between these contigs and rodent hepacivirus were 0.16 and 0.15, they were identified as variants of rodent hepacivirus.

#### Reoviridae

**Figure 7:**
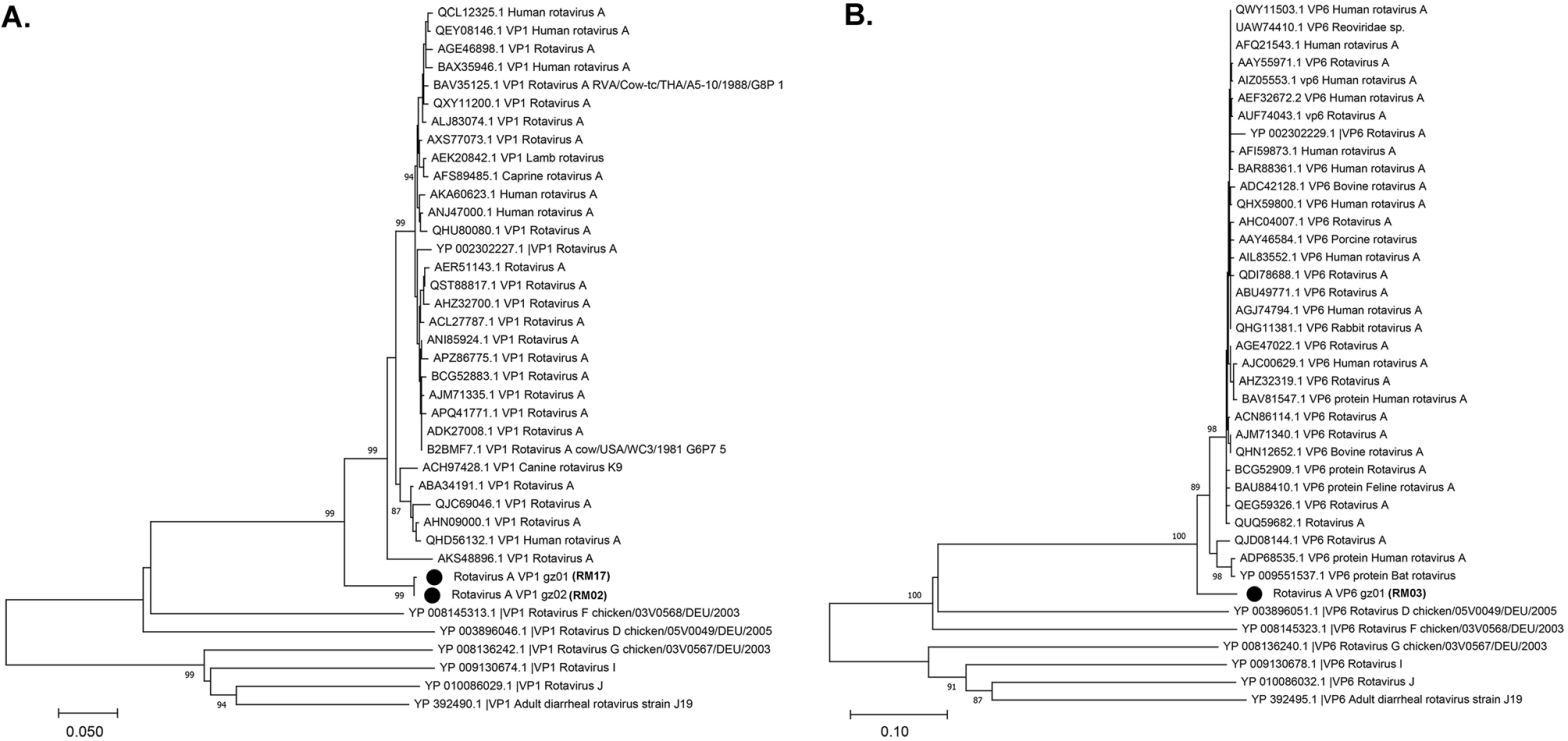
Phylogenetic relationships of vole rotavirus A based on the VP1 (RdRp) protein (**A**) and VP6 protein (**B**). Branch lengths are drawn to a scale of aa substitutions per site. Numbers above individual branches indicate bootstrap support, only values > 80% are shown. Novel rotavirus A variants are marked by a black dot, sample ID were labeled in parentheses.

In the liver, spleen, intestine, and fecal pools, 16 near-complete or partial genome sequences (0.2–3.3 k nt) of viruses of the family *Reoviridae* and genus *Rotavirus* were characterized. Analyses using NCBI ORF finder revealed a similar genome structure to *Reoviridae*, including the VP1, VP2, VP3, VP4, VP6, VP7, NSP2, and NSP3 segments^38,41,42^. BLASTn analyses of seven PCR-amplified segments (two of VP1, two of VP2, and three of VP3) revealed that vole rotavirus was related to other viruses from a range of host species, including *Lama guanicoe*, chicken, *Rhinolophus blasii*, *Microtus agrestis*, and human, with nt similarities of 70.81–76.78% and aa identify of 67.67–86.85% to the closest relatives in the VP1, VP2, and VP3 segments (Supplementary Table S4). These findings were confirmed by the phylogenetic analyses of the VP1 and VP6 segments. The contigs clustered with the species rotavirus A (Fig. 7). According to the aa sequence identities of the RdRp (VP1) and VP6 regions, these contigs were proposed to be novel variants or genotypes of rotavirus A^43^.

#### Picobirnaviridae

**Figure 8:**
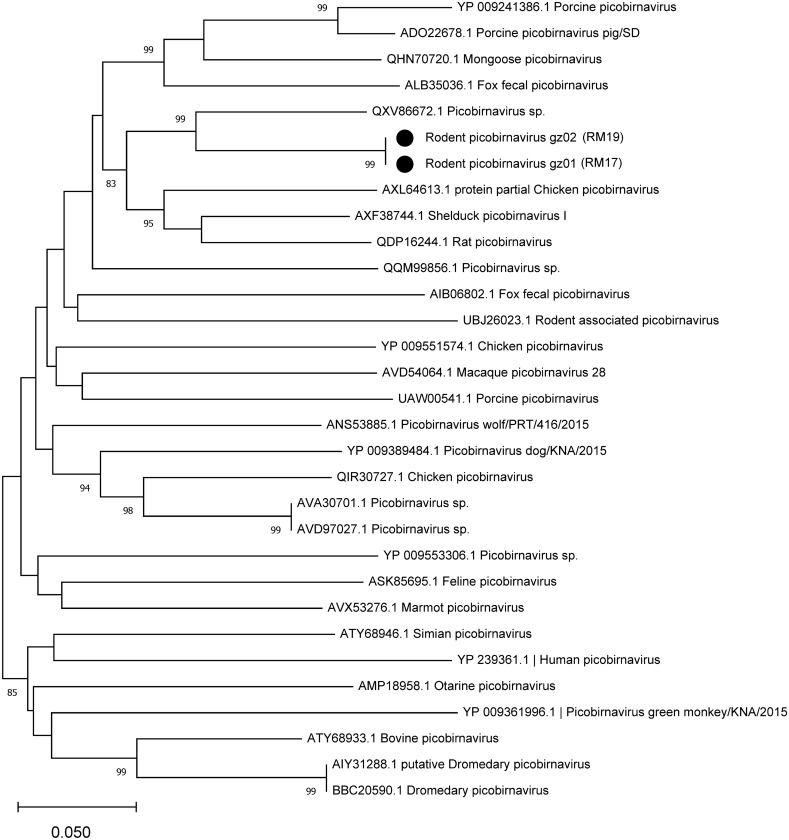
Phylogenetic analyses of picobirnavirus genomes on the basis of the segment 2 (RdRp) aa sequence. Branch lengths are drawn to a scale of aa substitutions per site. Numbers above individual branches indicate the bootstrap support, only values > 80% are shown. The novel variants of picobirnavirus are marked with a black dot, sample ID were labeled in parentheses.

In this study, 90% of picobirnavirus (PBV) sequence reads were detected in fecal samples. Two PBV contigs were obtained and PCR-confirmed from two fecal pools, with lengths of 1685/1684 bp. The distributions of these sequences were coincident with other PBVs, which have been detected in the feces of human, rabbit, dog, pig, rat, and bird^5,41^. Further analyses of these two segments revealed 2 RdRp region of PBV. These two segments showed low similarity to PBV sequences in GenBank. Based on the best RdRp matches from a BLASTn and BLASTx search, and several related strains from GenBank, nucleotide and protein phylogenetic trees were constructed separately. The two segments clustered with PBVs detected in fecal samples of rat collected in China, with 81.4% nt identity and 81.2% aa identity, respectively (Fig. 8 and Supplementary Table S4). According to the ICTV guidance, the high similarly between RdRp and Rat PBV revealed that these segments are new variants of PBV^44^.

#### Other potential mammalian viruses

Other sequence reads or contigs related to mammalian viruses showed low nucleotide and amino acid sequence identities (< 80%) with known viruses. Of 13 vertebrate-associated viruses identified, 9 were selected (Supplementary Table S4) for confirmation by PCR screening and Sanger sequencing. In addition to hepatovirus, hepacivirus, rotavirus, and PBV, astrovirus were verified in fecal samples. The assembled Astrovirus contigs with length of 242–343 bp showed 69–78.9% nt identity and 64.6–76.3% aa identity to diverse Astrovirus.

Moreover, some sequence reads related to the families *Coronaviridae*, *Circoviridae*, *Parvoviridae*, and *Arteriviridae* were occasionally detected and confirmed by RT-PCR. However, these segments were too short to identify genotypes, this suggests that these viruses might be of low viral load. Among them, coronavirus contigs were detected only in the fecal library (275 and 249 bp), and showed similarity to a known rodent coronavirus strain, Lucheng Rn rat coronavirus (MT820627.1), belonging to the genus *Alphacoronavirus*, with 87.94% nt identity and 91.21% aa identity. The circovirus contigs from the intestine and fecal libraries (367 bp) showed 78.11% nt identity to a feline cyclovirus (KM017740.1). Some contigs related to the family *Parvoviridae* were also identified, showing 74.86% similarity at the nt level and 73.75% at the aa level to a murine bocavirus (NC_055487.1). Sequence reads of *Arteriviridae* were identified in lung and spleen, one contig was retrieved from spleen (350 bp) showed 70.6% nt identity and 73.9% aa identity to Arteriviridae sp., which was detected in *Mus pahari* in Thailand (MT085142) (Supplementary Table S4).

## Discussion

We evaluated the viral spectrum in the tissues of 41 wild Qinghai voles, a typical wild rodent on the eastern Tibetan Plateau, and explored the phylogenetic relationships of several viruses of interest for human health. Overall, we identified 1,472,071 viral-associated sequence reads of 46 viral families known to infect vertebrates, invertebrates, plants, and other hosts. The viromes were dominated by vertebrate viral sequences (81.9% viral sequence reads were assigned to 13 viral families known to strictly infect vertebrates). Nevertheless, the smaller number of invertebrate and plant virus sequences indicated high viral diversity, accounting for 22 families. Among them, several sequences of plant and insect viruses also occurred in intestine or fecal samples demonstrating the omnivorous diet of the rodents, consistent with prior reports^9,12^. Furthermore, unclassified viruses were observed in each pool, particularly in intestine and fecal samples, accounting for 0.01–7.06% of total viral-related sequencing reads. These sequences distantly related to known viral sequences were of unknown taxonomic origin, which suggests that our knowledge of rodent viral variation is limited and that further research is needed^11,17^.

Although we detected no human viral pathogen sequences, some sequences had high similarity to known rodent viruses. The identification of novel variants of rodent viruses provides insight into the origin or evolution of important human or animal pathogens. Among them, *Hepatovirus* belongs to the family of Picornaviridae, which are small, nonenveloped, linear ssRNA (+) genome of 7.1–8.9 kb, polyadenylated, composed of a single ORF encoding a polyprotein. Human and vertebrates are natural hosts of Picornavirus and liver is the primary site of infection^13,36^. It’s transmission mainly depends on fecal-oral or blood route. Diverse picornaviruses cause mucocutaneous, encephalic, cardiac, hepatic, neurological, and respiratory diseases in a wide variety of vertebrate hosts^34,35^.

Moreover, *Hepaciviruses* constitute a genus that belongs to the positive, single-stranded RNA virus family of Flaviviridae. Their genome is approximately 10 kb long and encodes a single ORF translated into a polyprotein, which is processed by viral and cellular proteases, giving mature proteins^16,38,39^. These viruses infect a variety of mammalian hosts, such as primates, bats, horses, and rodents^45,46^. The main mode of transmission of *Hepaciviruses* is vertical, but the fecal route for cross-species transmissions has been suggested^39^. Genus Rotavirus belongs to the family of *Reoviridae*, which is non enveloped, segmented linear dsRNA genome virus. There are 11 segments coding for 12 proteins in its genome, size range from 0.6 to 3.3 k nucleotide. Rotaviruses exhibit substantial host-range restriction, including human and other vertebrates^42^. It’s transmission primary trough fecal-oral rote, was an important zoonosis^38,41,42^. PBV is small, nonenveloped viruses with a segmented linear dsRNA genome, segments size ranges from 1.7 to 2.5 kb. In which, the large segment encodes a capsid preceursor, while the small one encodes the viral RNA-dependent RNA polymerase (RdRp)^5^. PBVs have been detected in the feces of many different hosts, including humans, rabbits, dogs, pigs, rats, and birds^5,41^. PBVs can infect hosts and cause diarrhea, and they are considered to be opportunistic gastrointestinal pathogens associated with clinical disease in humans^34^.

Despite the high similar of genome and tissue distribution characteristics between novel variants observed in wild voles and members of their own viral families. However, it is still difficult to accurately define the relationship between the host virome and disease. The virome composition in animals is alike in both healthy or pathogenic and can influence host interactions^13^. Because many viruses revealed by metagenomic analyses in our study showed limited identity with known viruses, the threat to human and animals is unknown and therefore needs further investigation^1,18^. Continuous epidemiological surveillance and molecular studies are critical for an understanding of adverse effects as well as the control and prevention of the transfer of rodent viruses to people and other animals.

During the lifetime of an animal, the virome varies in response to changes in the environment such as exposure to a new pathogen or changes in diet and individual characteristics, such as immune response or age^7,32^. Proper specimen collection is vital. Viral families, whether originating from vertebrates, invertebrates, or plants, are not evenly distributed among tissues^9,47^. However, few studies have investigated viral populations in different tissues. Many animal pathogens are transmitted by the respiratory or oral-fecal route, so previous studies have focused on viral communities in respiratory or fecal samples, which are easy to collect noninvasively. However, a sample must be collected during virus shedding. Similarly, the collection of blood samples will yield virus sequences only in cases of a viremia, causing potential problems in latency or oscillating patterns of viremia during persistent infections^36^. It would be worthwhile to investigate different sample types, including feces, skin, urine, saliva, and tissues or organs, to assess viral diversity^9^. This would help determine viral tissue tropism and provide insight into routes of transmission^7^. The identification of viruses in environmental samples raises the question of whether these viruses are harbored by the host animal and cause infection, or merely infect insects, fungi, plants, or other environmental sources associated with the host’s life habits^32^.

We used metagenomics to compare the viral distribution in the liver, lung, spleen, intestine, and feces of wild Qinghai voles. Among them, the lung is often infected by respiratory viruses, it is also the site where viral spillover initiates between species such as influenza virus, adenovirus, rhinovirus, and coronavirus^17^. The spleen and liver are reservoirs of bloodborne viruses, such as dengue virus, West Nile virus, and arbovirus^5,47^. Further, small intestine and fecal samples are used to screen for oral-fecal pathogens, such as enteroviruses^48,49^. Our results show that the core virome of rodent tissues is different from those obtained from nasopharyngeal or rectal areas^7,36^. This difference may be due to the different viral tissue tropisms and transmission routes. The widely distributed RNA viruses detected in this study, such as hepacivirus and arterivirus, were not or only occasionally found in oral and anal samples in prior works^7,11^. By contrast, viruses transmitted by the fecal-oral or respiratory route, such as PBV, rotavirus, adenovirus, coronavirus, circovirus, and parvovirus, were seldom or never detected in internal tissue samples in this study. The presence of numerous non-vertebrate viral sequences in the intestine and fecal virome complicates the definition of the host-virus relationships, as observed by others^12^. Hence, tissue-based virome analysis is crucial for accurate assessment of viral infection status in natural reservoirs^17^. It is worth noting that, to collected tissue samples, break-back traps were used in this study, which might emerge potential complication results. Although we did not observe internal damage to organs, it is difficult to completely avoid the affects by compartment disruption and in vitro environmental factors. To minimize potential contamination, the inner layer of tissue and middle part of intestine with contents were collected in our study. Compare with other studies, which collected throat swabs, anal swabs or in vitro fecal samples, our results are less influenced by the external environment. Our findings provide insight into threats from infections of natural hosts and from the environment.

In contrast with other studies, relatively the small sample size and analysis of a single species were limitations of this study. However, we aimed to characterize viral diversity in a unique rodent species in the eastern Tibetan Plateau, and focused on the virome of the Qinghai vole, which is the most common rodent species and has the closest association with people and livestock in this area. The monitoring of rodent viromes will require extensive geographical and more diverse species sampling in the future. In addition, some sequence reads were identified as *Coronaviridae*, *Circoviridae*, *Parvoviridae*, and *Arteriviridae*. However, it was failed to identify the genotype of these viruses. This result may be due to a low viral load or spurious sequence similarities, as in a prior metagenomic study^17^. The characteristics of these viruses require further exploration. Furthermore, although the host genome sequences were filtered out from the dataset by mapping to the vole reference genome, the possibility cannot be excluded that some of these sequences were derived from endogenous viral sequences, which are viral sequences integrated into the animal genome^50^. At last, we noticed that viral reads of sample RM02 was significantly higher than others (2.47%). After remove this sample from analysis, the proportion of vertebrate viruses became 66.4%, liver and feces reserve viral reads were 18.5% and 47.5%, respectively. The proportions have changed, but the main conclusions were not affected by this sample.

A high proportion of zoonoses originate from wildlife reservoirs, and wild rodents are primary natural reservoirs for zoonotic pathogens^51^. Because of their health and economic impact, there is a growing awareness of rodent vector zoonotic infections, particularly viruses^34^. The transmission of rodent-associated viruses might result in regional disease outbreaks and global pandemics. Hence, a better understanding of virome diversity in rodents is important. Over the past decade, viral metagenomic analyses have expanded the known viral diversity of rodents^2,47,48,50,52^. These findings suggest that further viral discovery studies in rodent populations may benefit public health^53,54^. However, data on the viral richness of wildlife are sparse, and missing viruses merit further systematic surveillance globally, including in primates, bats, and rodents^55–57^.

In the plateau pastoral area of the Eastern Tibetan Plateau, Qinghai voles are unique and the most common rodent species, as well as important hosts for pathogens that transmit diseases to people and domestic animals because of their close association with both. Our analyses of the viromes of these voles expands knowledge of viral host range and evolution and will facilitate further studies that seek to identify changes in animal viromes and novel human infectious diseases before they emerge. However, there are still many gaps on rodents’ viral diversity which revealed by this study, it is necessary to enhance surveillance of virome diversity in rodents to facilitate prevention and control of emerging zoonotic diseases.

## Methods

### Rodent collection

In late August, 2020, rodent collection was conducted at Yunbo Gou in northwestern Shiqu County (Ganze Tibetan Autonomous Prefecture, Sichuan Province, China) with an elevation of 4200–4700 m above sea level. Grassland was the main vegetation type, covering > 90% of the collection area. Rodents were trapped using break-back traps set at the entrance of their dens, and species were identified by morphological characteristics. A total of forty-one Qinghai voles were used in this study. The location and topographic information were noted in maps generated by online software SuperMap (http://www.supermapol.com/).

More than 90% of individuals died immediately after captured. Captured survivors were anesthetized with ethyl ether then artificially killed and soaked in 75% ethanol for surface sterilization. Then tissue samples including lung, liver, spleen, and small and large intestines (with contents) were collected with disposable dissection instruments in the field. Each sample was preserved in independent sterile sample tubes, temporarily stored at –20 °C, and transported to the laboratory in Beijing and stored at –80 °C. The heads of the rodents were stored in 95% ethanol in a 50 mL capped tube for further species confirmation. All sampling work was conducted during the annual wildlife parasite surveillance, conducted by China CDC. Sample collection and all experiments in the present study were performed with ethical approval given by the Ethics Committee of National Institute for Parasitic Diseases, Chinese Center for Disease Control and Prevention (reference number IPD-2021-15). All methods were performed in accordance with the relevant guidelines and regulations and reported in accordance with recommendations in the ARRIVE guidelines.

### Viral nucleic acid extraction, reverse transcription and cDNA amplification

The inner layer of tissue and fecal samples (20–30 mg) was disrupted and homogenized using a TissueLyser (Qiagen) in the presence of lysis buffer (Buffer RLT Plus, Qiagen) and stainless-steel beads (5 mm mean diameter). Viral RNA was extracted from each lysate using QIAamp Viral RNA Mini Kit (Qiagen) according to the manufacturer’s protocol. RNA was eluted to a final volume of 50 µL and stored at −80 °C until further use. Purified RNA was quantified using a Nanodrop (Thermofisher), and RNA from the same tissue was pooled in equal quantities, resulting in 20 pools. Each pool, at the tissue level, included 10–11 individuals according to sample availability. Overall, 205 tissue and fecal samples from 41 individuals were analyzed (Supplementary Table S1).

Prior to processing, rRNA was removed from each pool using the QIAseq FastSelect rRNA Remove Kit (Qiagen). SuperScript IV Reverse Transcriptase (Invitrogen) with Random Hexamer primer was used to reverse transcribe RNA into cDNA. DNA polymerase I (NEB) was next added to synthesize the second strand of cDNA. For each RNA pool, the whole genome was amplified from cDNA by the multiple displacement amplification (MDA) method (Qiagen). Amplified products were purified using beads (AMPure XP system) and solubilized in 30 µL TE buffer. DNA concentration was measured using a Qubit dsDNA Assay Kit in a Qubit 3.0 Flurometer (Life Technologies). The resulting dsDNA products were used to construct 20 sequencing libraries.

### RNA library construction and next-generation sequencing

The sequencing libraries were generated using the Nextera XT DNA Sample Preparation Kit (Illumina) following the manufacturer’s recommendations. Briefly, the DNA samples were fragmented and ligated with the adaptor for PCR amplification. PCR products were purified and quantified. Sequencing was carried out by Novogene Company (China) on the Illumina Novaseq platform in 150 bp paired ends with dual barcoding for each pool.

### Bioinformatics analyses of viral metagenomic sequencing

An in-house analysis pipeline was used to process the data, and a flowchart is shown in Supplementary Fig. S1, drawn using edraw (edrawsoft, v. 10.1.7). First, quality control was conducted using fastp (v. 0.20.1) software^58^. By default, unqualified reads (low quality, too short, or too many N reads) were excluded and total numbers of clean reads were obtained from 20 pools, then they were mapped against the vole reference genome in the NCBI database using Bowtie2 (v. 2.4.4) to determine the host background percentage^59^.

All unaligned reads were de novo assembled with MEGAHIT (v. 1.2.9) using default parameters, and the minimum contig length was set at 151 nucleotides^60^. Contigs were clustered to remove duplicate sequences using Cd-hit (v. 4.8.1) software^61^. The resulting unique contigs were submitted to BLASTx against the entire NCBI-nr database (December 2020) using DIAMOND (v. 2.0.6.144)^62^. Any contigs belonging to known cellular organisms (bacteria, archaea, and eukaryotes) were excluded, and virus-specific contigs and unclassified contigs were obtained to construct a unique dataset. Previously cleaned data were mapped back to the unique dataset using Bowtie2 and SAMtools (v. 1.12) to generate viral related dataset for each pool^63^.

Virus taxonomic assignment was further conducted based on sequence similarity. The viral related dataset generated in the previous step were again proceed using DIAMOND with comparisons drawn to a viral protein database (NCBI virus, July 2020) for classification to the lowest taxonomic unit possible. Based on the BLASTx output, sequences were classified as viral families based on the taxonomic origin of the best-hit (lowest E score) sequence match. An E value of 10^–10^ was the cutoff value for significant hits. To detect potential viruses with remote similarities, each pool of sequences classified as viruses was de novo assembled into contigs by metaSPAdes (v. 3.11.1) and subjected to BLASTn (v. 2.10.1) or BLASTx search against the virus nucleotide and amino acid database (NCBI virus, July 2020), respectively^64,65^. The taxonomies of the aligned reads with the best BLAST scores were parsed using the MEGAN6 Metagenome Analyzer (v. 6.21.10) with the lowest common ancestor (LCA) approach ^66^. While maintaining a low false-positive rate, final taxonomy reports were generated by combining BLASTn and BLASTx virus classification entries for pairwise comparisons.

To more accurately define taxonomic status, each viral taxonomic identification was associated with host types, such as bacteria, vertebrates, invertebrates, amoeba, and plants, by consulting the International Committee on Taxonomy of Viruses (ICTV) report and ViralZone database^67,68^. Based on the numbers of viral reads and the abundance information in each pool at the viral family level, statistical analyses and visual display were performed using GraphPad Prism (v.9.0.0, GraphPad Software, San Diego, California USA, www.graphpad.com) and Hiplot (v0.2.0, https://hiplot.com.cn).

### PCR verification and phylogenetic analyses of selected virus

All assembled viral contigs and any viral-like contigs classified into members of zoonotic viruses or rodent-associated viruses such as *Reoviridae*, *Coronaviridae*, *Flaviviridae*, *Picornaviridae*, and *Parvoviridae* were extracted. The nucleotide and amino acid identities between these contigs and reference sequence were determined using the alignment results from BLASTx or BLASTn. Specific primers were designed from the contigs to screen for each virus in the individual pools. The presence of viruses was confirmed by PCR amplification using specific primers. All related reference viral sequences were downloaded from GenBank, and contigs were aligned against their reference sequences to indicate their corresponding genomic locations and relative genetic distances. Potential open reading frames (ORFs) were identified using ORF Finder (https://www.ncbi.nlm.nih.gov/orffinder/). The genome or contigs of each virus were aligned with representative amino acid sequences or segments (alignment regions were chosen according to the ICTV guidelines for each virus) using MAFFT (v. 7.487), and phylogenetic analyses were undertaken using the neighbor-joining method of maximum composite likelihood with the bootstrap set to 1,000 repeats in MEGA-X (v. 10.2.1)^69,70^.

## Supplementary Information

Supplementary Information 1.

Supplementary Information 2.

Supplementary Information 3.

Supplementary Information 4.

Supplementary Information 5.

Supplementary Information 6.

Supplementary Information 7.

## Data Availability

The datasets generated and analyzed in this study are available in this published article (and its supplementary information files). Illumina Novaseq sequence data was deposited into NCBI BioProject under accession number PRJNA776963 (https://www.ncbi.nlm.nih.gov/bioproject/PRJNA776963). All virus sequences reported in this study were deposited in the GenBank nucleotide database (https://www.ncbi.nlm.nih.gov/genbank/) under accession numbers OL401529–44 and OL342359–65 (Supplementary Table S4).
