# Supplementary figures and images for: Metagenomic analysis of viromes in tissues of wild Qinghai vole from the eastern Tibetan Plateau
Source: Sci Rep. 2022 Oct 14;12:17239. doi: 10.1038/s41598-022-22134-y (PMC9562062; doi:10.1038/s41598-022-22134-y)

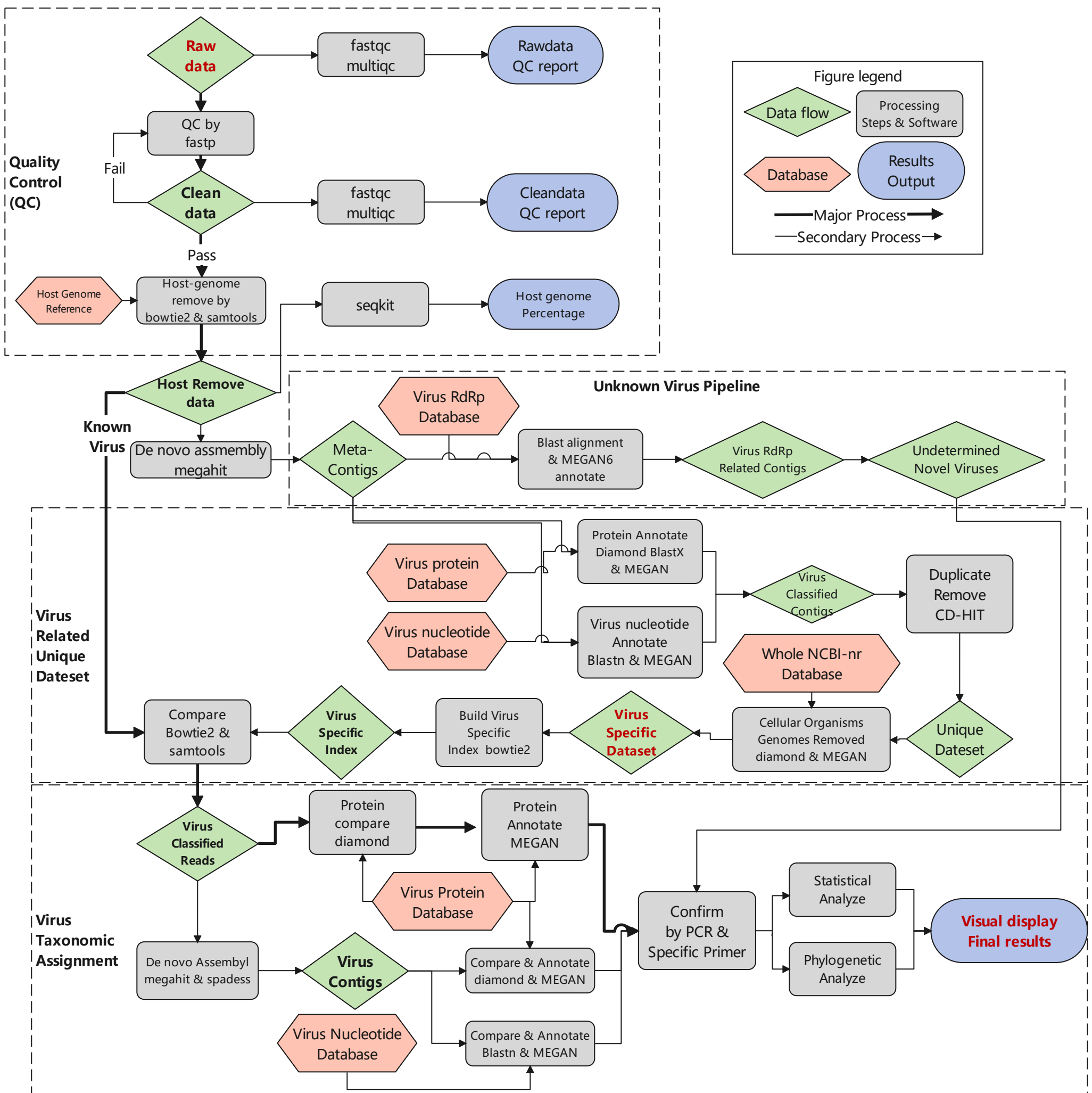

Supplement: Supplementary file 1 — Supplementary Information 1. [file 41598_2022_22134_MOESM1_ESM.pdf]
