# Supplementary material for: Metagenomic analysis of viromes in tissues of wild Qinghai vole from the eastern Tibetan Plateau
Source: Sci Rep. 2022 Oct 14;12:17239. doi: 10.1038/s41598-022-22134-y (PMC9562062; doi:10.1038/s41598-022-22134-y)
Supplement: Supplementary file 7 — Supplementary Information 7. [file 41598_2022_22134_MOESM7_ESM.docx]

**Figure S1. Flowchart of virus identification using metagenomic sequencing data.** The steps included quality control of rawdata, construction of a unique virus-related dataset, virus taxonomic assignment, and analysis of unknown viruses.

**Figure S2. Phylogram of identified viral families using MEGAN 6.**
