# Supplementary material for: Metagenomic analysis of viromes in tissues of wild Qinghai vole from the eastern Tibetan Plateau
Source: Sci Rep. 2022 Oct 14;12:17239. doi: 10.1038/s41598-022-22134-y (PMC9562062; doi:10.1038/s41598-022-22134-y)
Supplement: Supplementary file 2 — Supplementary Information 2. [file 41598_2022_22134_MOESM2_ESM.pdf]

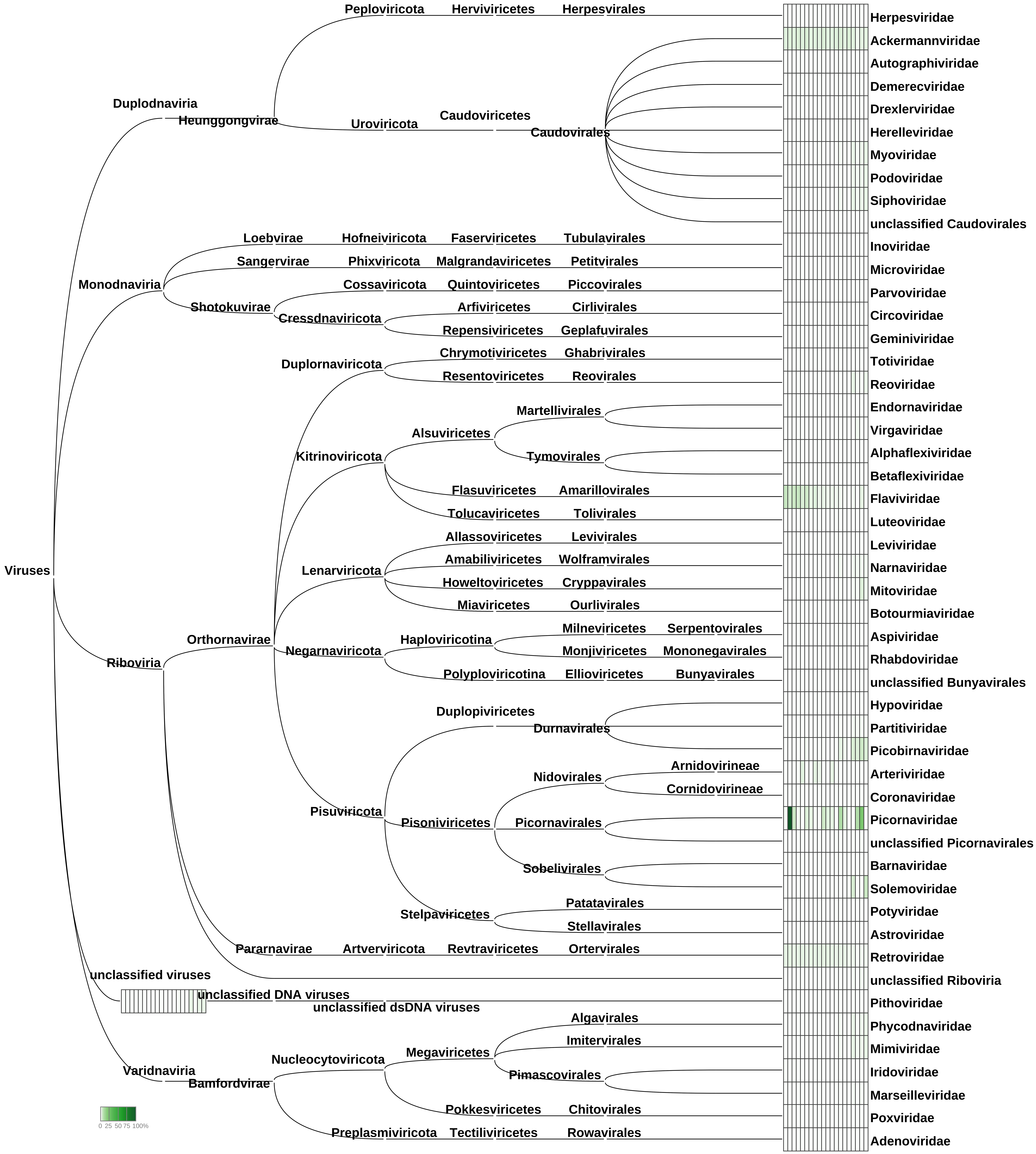
Viruses
Duplodnaviria
Heunggongvirae
Peploviricota
Herviviricetes
Herpesvirales
Uroviricota
Caudoviricetes
Caudovirales
Monodnaviria
Loebvirae
Hofneiviricota
Faserviricetes
Tubulavirales
Sangervirae
Phixviricota
Malgrandaviricetes
Petitvirales
Shotokuvirae
Cossaviricota
Quintoviricetes
Piccovirales
Cressdnaviricota
Arfiviricetes
Cirlivirales
Repensiviricetes
Geplafuvirales
Riboviria
Orthornavirae
Duplornaviricota
Chrymotiviricetes
Ghabrivirales
Resentoviricetes
Reovirales
Kitrinoviricota
Alsuviricetes
Martellivirales
Tymovirales
Flasuviricetes
Amarillovirales
Tolucaviricetes
Tolivirales
Lenarviricota
Allassoviricetes
Levivirales
Amabiliviricetes
Wolframvirales
Howeltoviricetes
Cryppavirales
Miaviricetes
Ourlivirales
Negarnaviricota
Haploviricotina
Milneviricetes
Serpentovirales
Monjiviricetes
Mononegavirales
Polyploviricotina
Ellioviricetes
Bunyavirales
Pisuviricota
Duplopiviricetes
Durnavirales
Pisoniviricetes
Nidovirales
Arnidovirineae
Cornidovirineae
Picornavirales
Sobelivirales
Stelpaviricetes
Patatavirales
Stellavirales
Pararnavirae
Artverviricota
Revtraviricetes
Ortervirales
unclassified viruses
unclassified DNA viruses
unclassified dsDNA viruses
Varidnaviria
Bamfordvirae
Nucleocytoviricota
Megaviricetes
Algavirales
Imitervirales
Pimascovirales
Pokkesviricetes
Chitovirales
Preplasmiviricota
Tectiliviricetes
Rowavirales
Herpesviridae
Ackermannviridae
Autographiviridae
Demerecviridae
Drexlerviridae
Herelleviridae
Myoviridae
Podoviridae
Siphoviridae
unclassified Caudovirales
Inoviridae
Microviridae
Parvoviridae
Circoviridae
Geminiviridae
Totiviridae
Reoviridae
Endornaviridae
Virgaviridae
Alphaflexiviridae
Betaflexiviridae
Flaviviridae
Luteoviridae
Leviviridae
Narnaviridae
Mitoviridae
Botourmiaviridae
Aspiviridae
Rhabdoviridae
unclassified Bunyavirales
Hypoviridae
Partitiviridae
Picobirnaviridae
Arteriviridae
Coronaviridae
Picornaviridae
unclassified Picornavirales
Barnaviridae
Solemoviridae
Potyviridae
Astroviridae
Retroviridae
unclassified Riboviria
Pithoviridae
Phycodnaviridae
Mimiviridae
Iridoviridae
Marseilleviridae
Poxviridae
Adenoviridae
0 25 50 75 100%
